# A National Survey of Musculoskeletal Impairment in Rwanda: Prevalence, Causes and Service Implications

**DOI:** 10.1371/journal.pone.0002851

**Published:** 2008-08-06

**Authors:** Oluwarantimi Atijosan, Dorothea Rischewski, Victoria Simms, Hannah Kuper, Bonaventure Linganwa, Assuman Nuhi, Allen Foster, Chris Lavy

**Affiliations:** 1 Department of Infectious and Tropical Diseases, London School of Hygiene & Tropical Medicine, London, United Kingdom; 2 CBMI, Kigali, Rwanda; 3 Nuffield Department of Orthopaedic Surgery, University of Oxford, Oxford, United Kingdom; University of Otago, New Zealand

## Abstract

**Background:**

Accurate information on the prevalence and causes of musculoskeletal impairment (MSI) is lacking in low income countries. We present a new survey methodology that is based on sound epidemiological principles and is linked to the World Health Organisation's International Classification of Functioning.

**Methods:**

Clusters were selected with probability proportionate to size. Households were selected within clusters through compact segment sampling. 105 clusters of 80 people (all ages) were included. All participants were screened for MSI by a physiotherapist and medical assistant. Possible cases plus a random sample of 10% of non-MSI cases were examined further to ascertain diagnosis, aetiology, quality of life, and treatment needs.

**Findings:**

6757 of 8368 enumerated individuals (80.8%) were screened. There were 352 cases, giving an overall prevalence for MSI of 5.2%. (95% CI 4.5–5.9) The prevalence of MSI increased with age and was similar in men and women. Extrapolating these estimates, there are approximately 488,000 MSI diagnoses in Rwanda. Only 8.2% of MSI cases were severe, while the majority were moderate (43.7%) or mild (46.3%). Diagnostic categories comprised 11.5% congenital, 31.3% trauma, 3.8% infection, 9.0% neurological, and 44.4% non-traumatic non infective acquired. The most common individual diagnoses were joint disease (13.3%), angular limb deformity (9.7%) and fracture mal- and non-union (7.2%). 96% of all cases required further treatment.

**Interpretation:**

This survey demonstrates a large burden of MSI in Rwanda, which is mostly untreated. The survey methodology will be useful in other low income countries, to assist with planning services and monitoring trends.

## Introduction

There is a global lack of accurate information on the prevalence and causes of physical disability in low income countries [1], [2]. There are two main reasons for this deficiency. Firstly, there have not been many surveys and secondly there is no universally accepted definition of physical disability. The surveys that have been undertaken have used a variety of definitions of physical disability, and a range of methodologies for measuring disability so that comparisons cannot be made between countries [3], [4] . For example one survey which asked detailed questions about difficulties in different aspects of life, showed that Norway had a prevalence of physical disability of 35% while the national census in India, which merely asked whether there was a “physically handicapped” person in the household estimated that the prevalence was 0.2% [5]. With such different ways of measuring and defining disability there is little benefit in making comparisons between countries, or over time within a country. Where there has been a tighter definition of a specific impairment or symptom such as has been used in the COPCORD programme (Community-Oriented Program for the Control of Rheumatic Diseases) [6] then it has been possible to standardise a data collection methodology, with scope for international comparison. The COPCORD programme is of great value in comparing rheumatic and joint conditions in different countries, however it does not include trauma or non painful congenital or acquired musculoskeletal deformities.

The difficulty in defining physical disability stems from its many anatomical, physiological and pathological presentations and causes, and its intimate relation to society and the environment. Terminology has also been confusing, and different groups in society have different reasons for the varied used of the word disability. This debate is of more than just academic interest as in order to plan effective services it is important to estimate the prevalence and causes of physical disability, which requires a definition of the disability being measured and a survey methodology. There have been many attempts to reach a common understanding of disability, and the World Health Organisation's (WHO) publication of the International Classification on Functioning (ICF) is a major step forward [7]. The ICF classifies impairment of body structure and function, and also includes domains that measure activity and participation in society.

Rwanda as a country is in the process of rebuilding its rehabilitation services and facilities for people with musculoskeletal impairment (MSI) after its genocide and war of 1994 with all the demographic and structural destruction that took place. In order to plan effective services it is important to estimate the prevalence and causes of MSI that exist in the country. The WHO estimates that the prevalence of all types of disability on a global level is around 10% [8], but this estimate is of limited use for planning services in specific situations. Realising this difficulty, Helander developed a ‘Rapid Calculation of Disability Prevalence’ for less developed regions of the world and estimated that 4.8% of a population will need some rehabilitation service [9]. Several physical disability surveys have been conducted in Rwanda since the 1994 war, but all have their limitations. Handicap International carried out a nationwide survey in 1995 into ‘physical disability’ [10], and found a very low prevalence of 0.58%. Its own researchers noted that this was low and questioned whether many sections of the population might have been inaccessible so soon after the war. A Community Based Rehabilitation project in Kigali carried out a similar survey in 1997 and estimated that the prevalence was 1.8% (personal communication), but the sampling methodology was inadequate, and the researchers believed that many households withheld information about family members with physical disabilities.

In view of the lack of accurate data on the prevalence and causes of MSI, we worked with the Ministry of Health of Rwanda to develop a survey of MSI of all ages that involved a reliable sampling methodology and a case definition and diagnostic criteria that could clearly map onto the classification system used in the ICF. Our aim was to develop a reliable survey tool that could be used to plan and monitor MSI services in Rwanda and other developing countries.

## Methods

### Sample selection – (see also diagram in Appendix S1)

The survey was designed to be nationally representative, including people of all ages. The expected prevalence of MSI in this group was estimated at approximately 3% [9], [11]. Allowing for a required confidence of 95%, a precision of 20%, a population size of 8,441,000 in 2005 [11], a design effect of 2.3, and 15% non-response, the required sample size was estimated to be 8399 subjects (Epi Info 6.04). In total, 105 clusters of 80 people were needed for this survey. A cluster size of 80 people was chosen for logistical reasons, as it was considered to be the number a team could complete in one day.

A nationally representative sample of the population was selected through cluster sampling with probability proportionate to size. A list was produced of all the enumeration areas and their respective populations, and a column was created with the cumulative population across the settlements. The total population (i.e. 8,441,000) was divided by the number of clusters required (i.e. 105) to derive the sampling interval (i.e. 80,390). The first cluster was selected by multiplying the sampling interval with a random number between 0 and 1. The resulting number was traced in the cumulative population column and the first cluster was taken from the corresponding enumeration area. The following clusters were identified by adding the sampling interval to the previous number.

Households within clusters were selected through compact segment sampling [12]. Maps of each selected cluster (i.e. enumeration area) were obtained from the census bureau. These maps included the locations of the head of ten-household communities, thus showing approximate population distribution. The enumeration area was visited two to three days before the survey and the village leaders were asked to update the map. The enumeration area was then divided into segments, so that each segment included approximately 80 people. For instance, if an enumeration area comprised 400 people then it would be divided into five segments. One of the segments was chosen at random by drawing lots and all households in the segment were included in the sample sequentially until 80 people were identified. People were eligible for inclusion if they lived in the household at least three months of the year. All the individuals in the final household were screened, and the number of people needed to complete the cluster was randomly selected for inclusion (e.g. if the final household included 5 people but only 3 were required to complete the cluster then 3 out of the 5 were randomly selected for inclusion). If the segment did not include 80 people then another segment was chosen at random and sampling continued. If an eligible person was absent the survey team returned to the household to examine him/her before leaving the area. If after repeated visits the person could not be examined, information about his/her presumed MSI status was collected from relatives or neighbours.

### Musculoskeletal impairment assessment

The fieldwork was carried out between October and December, 2005. The survey team visited households door-to-door and conducted the MSI screening in the household. The survey team consisted of a physiotherapist and an assistant, and they were assisted in the clusters by a village guide, appointed by the village leaders. The purpose of the study and the examination procedure were explained to the subjects and verbal consent was obtained before examination.

A standardised protocol was used for the screening and assessment of MSI [13]. A survey record was filled for each eligible person that included:

- Demographic information (all participants);
- A screening examination for MSI (all participants);
- A standardised interview and examination protocol for MSI (cases and 10% random sample of non-cases)
- History of MSI (if not examined)

### Screening for musculoskeletal impairment

The team physiotherapist screened all participants for MSI by asking them seven questions about difficulties using their musculoskeletal system, whether they used a mobility aid, whether they felt they had any physical deformity, and how long they had had these symptoms. Participants over 5 years of age were questioned directly, while participants under 5 years were asked through proxy, by the child's main carer. Participants who answered “yes” to any of the questions were classified as cases, provided that the condition had lasted for more than one month or was considered permanent. This screening tool was developed by orthopaedic surgeons together with physiotherapists and has been shown to have 99% sensitivity and 97% specificity with interobserver Kappa scores of 0.90 for the diagnostic group [13].

### Standardised interview and examination protocol

All cases were examined in more detail by the physiotherapist using a standardised interview and examination protocol. A random 10% sample of non-cases was also examined further, to confirm their non-case status. The standardised examination protocol assessed the aetiology, duration, severity, anatomical location, diagnosis, and treatment, both received and required.

The standardised interview and examination protocol included the following sections:

a) Physical examination

The physiotherapist observed the participant as they carried out physical tasks that required use of the musculoskeletal system (i.e. walking, crouching and upper limb motor skills)

b) Diagnosis

The physiotherapist categorized the diagnosis as: congenital, traumatic, infective, neurological, or acquired non traumatic non infective. Within these categories an algorithm was used to give a specific diagnosis. Up to two diagnoses were permissible per identified case of MSI [13].

c) Area affected and nature of problem

The physiotherapist recorded information on the area of the body affected (e.g. arm) and the nature of the problem (e.g. amputation).

d) Aetiology

Where this was known it was recorded. It was determined by questioning the case about when the impairment developed and how it came about. The physiotherapists were trained as to what questions to ask for each aetiology available, which included road traffic accidents, war, infection, and familial.

e) Severity

Severity was determined using ICF parameters for the amount of function which has been lost through the presence of the MSI. This was classified as “mild”, “moderate” or “severe” [6].

f) EQ-5D

Generic quality of life was measured using the EQ-5D scale, which is a public domain health-related quality of life questionnaire [14]. This was translated and back translated from English into Kinyarwandan by two medical translators, independently of each other. However, because of time restrictions, this was carried out independently from the Euroqol group, and the translated version of EQ-5D used in this study has therefore *not* been approved by the Euroqol group

g) Treatment given

Current treatment received by the participant (if any) was recorded.

h) Barriers to treatment

Cases were asked an open-ended question about why they had not accessed treatment for their MSI. Up to four responses were recorded per case on pre-coded forms.

i) Treatment needed

Treatment needed was assessed by the physiotherapist according to standard protocols, appropriate for Rwanda.

### Training and quality control

There were three teams, each consisted of a physiotherapist a medical assistant, a village guide and a driver. The teams received three weeks of training. Inter-observer agreement for case definition, diagnosis, severity classification and treatment required was assessed between the teams to ensure that it was of an acceptable standard (i.e. kappa≥0.60). A pilot survey was undertaken of 480 people in 9 clusters (6 rural and 3 urban) to assess examination process and procedures. During the main survey, teams were accompanied by a field supervisor at least one day per week, to ensure that a high quality was maintained. Each day the supervisors checked items of all completed forms in the field.

### Statistical analysis

A database was constructed for data entry using EpiData 3.1. The data were double-entered and validated, and inconsistencies were checked. Stata 9.0 was used for analysis. The prevalence and causes of MSI was estimated, taking into account the design effect (DEFF) when estimating the confidence intervals. (see appendix S1 for details of estimation of DEFF)

### Ethical approval

Ethical approval for this survey was granted by the Independent Ethics Committee in Rwanda and the London School of Hygiene & Tropical Medicine. Permission to proceed was granted by the government, and consent was granted for each cluster visited from the community leader at the province, district, sector and cell level. Informed verbal consent was obtained from the subjects after explanation of the nature and possible consequences of the study. Written consent was obtained for any photographs that were taken. All people with treatable MSI were referred to a central community rehabilitation centre where clinical members of the study team reviewed and referred the participants for further treatment, as appropriate. The research followed the tenets of the Declaration of Helsinki.

*Role of funding source.*

*The funding for this study was provided by CBM international. One of the authors (OR) was supported by Cure International. The funding organisations played no part in, and had no influence on the design of the study, or the data, collection, analysis or interpretation.*

## Results

### Sampled population (table 1)

A total of 8368 individuals were enumerated and 6757 were screened (Response rate = 80.8%), 1596 (19.1%) were absent, 10 (0.1%) refused and 5(0.1%) were unable to communicate. The response rate was higher in women (84.8%) than in men (76.3%). Among the participants that were enumerated but not examined, 88 were believed to have MSI (5.5%). The age- and gender-distribution of the sampled population was very similar to that of the national population (Table 1).

**Table 1:** Age and gender composition of national* and screened sample population.

Age Groups	Male			Female			Total		
	49.7%	47%	44.3%	50.3%	52%	55.7%			
	National (%)	Enumerated Sample	Screened Sample (%)	National (%)	Enumerated Sample	S Screened ample (%)	National (%)	Enumerated Sample	Screened Sample (%)
**0–10**	1 302 000 (31.1)	1394 (35.4)	1222 (40.7)	1 287 000 (30.3)	1420 (32.1)	1295 (34.5)	2 589 000 (30.7)	2816 (33.7)	**2519 (37.3)**
**11–20**	964 000 (23.0)	1029 (26.2)	723 (24.1)	964 000 (22.7)	1081 (24.4)	832 (22.2)	1 929 000 (22.9)	2116 (25.3)	**1559 (23.1)**
**21–30**	807 000 (19.2)	601 (15.3)	386 (12.9)	808 000 (19.0)	724 (16.4)	567 (15.1)	1 616 000(19.1)	1325 (15.8)	**953 (14.1)**
**31–40**	482 000 (11.5)	335 (8.5)	234 (7.8)	467 000 (11.0)	422 (9.5)	358 (9.5)	949 000 (11.2)	757 (9.1)	**592 (8.8)**
**41–50**	326 000(7.7)	275 (7.0)	195 (6.5)	327 000 (7.7)	330 (7.5)	292 (7.8)	654 000 (7.7)	605 (7.2)	**487 (7.2)**
**51–60**	182 000(4.3)	160 (4.1)	126 (4.2)	205 000 (4.8)	231 (5.2)	203 (5.4)	387 000 (4.6)	392 (4.69)	**329 (4.9)**
**>60**	129 000 (3.1)	140 (3.6)	114 (3.8)	190 000 (4.5)	216 (4.9)	204 (5.4)	289 000 (3.4)	356 (4.3)	**318 (4.7)**
**Total**	**4 193 000 (100.0)**	**3934 (100.0)**	**3000 (100.0)**	**4 248 000 (100.0)**	**4424 (100.0)**	**3751 (100.0)**	**8 441 000 (100.0)**	**8367 (100.0)**	**6757** $ ** (100.0)**

* based on us census bureau data for Rwanda population 2005 as this has age group divisions

$ missing gender data for 6 individuals

### Prevalence of MSI

Of the 6757 individuals screened there were 352 cases of MSI giving an overall prevalence of MSI of 5.2% (CI 4.5–5.9) (Table 2). The prevalence of MSI fell after early childhood and then increased rapidly with age so that it was almost nine-fold higher in people aged over 60 years compared to those aged 0–5 years (OR = 8.9, 6.0–13.4). The prevalence of MSI was similar in men (5.1%) and women (5.3%). People in rural areas were more likely to have an MSI (5.4%) than urban dwellers (4.1%), while those without formal education were more likely to have an MSI (5.6%) than those with formal education (4.5%), although these associations disappeared after adjustment for age and gender.

**Table 2:** Prevalence of MSI by age, gender, location and educational level of head of household.

Categories*		total no screened.	No of MSI cases in that group	Prevalence of MSI (95% CI)	Age and sex adjusted Odds Ratios (95%CI)
	**Total**	**6757**	**352**	**5.2% (4.5–5.9%)**	
**Age groups**	0–5 years	1520	52	3.4% (2.3–4.5%)	1
	6–16 years	2006	39	1.9% (1.2–2.7%)	0.6 ( 0.4–0.9)
	17–60 years	2913	185	6.4% (5.3–7.4%)	1.9 (1.4–2.7)
	>60 years	318	76	23.9% (18.5–29.3%)	8.9 (6.0–13.4)
**Gender**	Male	3000	153	5.1% (4.3–6.0%)	1
	Female	3751	199	5.3% (4.5–6.2%)	0.9 (0.8–1.2)
**Location**	Rural	5806	312	5.4% (4.6–6.1%)	1
	Urban	938	39	4.1% (2.7–5.6%)	0.9 (0.6–1.4)
**Educational Level of Head of household**	No formal education	4346	244	5.6% (4.8–6.5%)	1
	Formal education	2399	108	4.5% (3.5–5.5%)	0.9 (0.7–1.2)

* There were some missing values

### Prevalence of MSI by Severity and Gender

The majority of cases of MSI were mild (47.1%) or moderate (44.5%), and few were severe (8.4%) (Table 3). This pattern was very similar in men and women.

**Table 3:** Distribution of MSI according to severity and gender, and its association with quality of life.

	Male		Female		Total		
MSI status	Number	Proportion of MSI cases	Number	Proportion of MSI cases	Number	Proportion of MSI cases	EQ-5D VAS Score (95% CI)
Mild MSI	69	46.0%	94	48.0%	163	47.1%	44.4 (40.5–47.8)
Moderate MSI	65	43.3%	89	45.4%	154	44.5%	37.7.(35.4–40.0)
Severe MSI	16	10.7%	13	6.6%	29	8.4%	16.9 (11.7–22.0)
No MSI	2847		3552		6399		63.1 (61.4–64.7)

### MSI Diagnoses

There were a total of 390 diagnoses for 352 people with MSI (Table 4). The most common causes of MSI were joint problems (13.3% of MSI diagnoses), other acquired (12.3%), fracture non or malunion (7.2%) and other chronic joint injury (6.2%). Overall 44% of MSI were due to acquired non-traumatic non-infective causes, 31% due to trauma, 9% neurological were in origin, 4% due to infection and 12% congenital. Extrapolating these estimates to the total population of Rwanda there were 488,000 MSI diagnoses.

**Table 4:** Cause of MSI in survey, and extrapolated to population of Rwanda.

	Diagnosis	Number	Total in category (%)	Extrapolated number of that diagnostic category in Rwanda to nearest 1000 (95%CI)
**A**	**Congenital deformity**		45 (12%)	59,000 (95% CI 39,000–74,000)
	Polydactyly	16		
	Syndactyly	2		
	Other upper limb deformity	4		
	Club foot	4		
	Other lower limb deformity	12		
	Spine deformity	1		
	Cleft lip or cleft palate	2		
	Multiple abnormalities	2		
	Other congenital deformity	2		
**B**	**Trauma**		122 (31%)	156,000 (95% CI 125,000–187,000)
	Fracture non or malunion	28		
	Burn contracture	4		
	Spine injury	3		
	Head injury	3		
	Joint chronic dislocation	6		
	Other chronic joint injury	24		
	Tendon, muscle or nerve injury	12		
	Amputation	20		
	Other traumatic MSI	22		
**C**	**Infective**		15 (4%)	20,000 (95% CI 9,000–29,000)
	Joint infection	4		
	Bone infection limb	8		
	Bone infection spine	1		
	Skin/soft tissue infection/wound	2		
**D**	**Neurological**		35 (9%)	44,000 (95% CI 27,000–60,000)
	Polio	8		
	Para/quadri/tetraplegia	11		
	Cerebral palsy or developmental delay	5		
	Peripheral nerve palsy	4		
	Other neurological MSI	7		
**E**	**Other acquired non-traumatic non-infective**		173 (44%)	216,000 (95% CI 182,000–245,000)
	Joint problem	52		
	Angular limb deformity	38		
	Skin/soft tissue tumour/swelling	12		
	Spine deformity	2		
	Spine pain	11		
	Limb pain	5		
	Limb swelling	5		
	Other acquired	48		
	TOTAL	390		488,000

**Figure 1:**
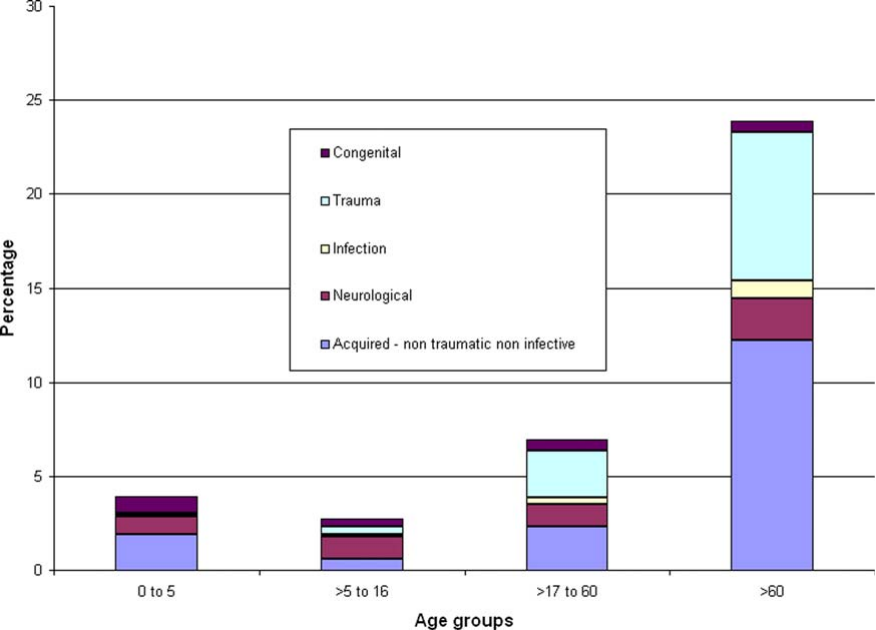
Prevalence and diagnostic categories of MSI, by age group.

With increasing age, the prevalence of MSI increased rapidly (Figure 1). The greatest proportional increase was in MSI diagnoses related to trauma and acquired non-traumatic non-infective. Congenital diagnoses were relatively more common in the youngest age group than in older people and the proportion of neurological diagnoses remained relatively constant with increasing age.

### Aetiology of MSI

The aetiology of almost one third (32.1%) of the cases was unknown. A further 28.1% were due to trauma, 15.1% due to infection, 11.4% due to family history. Other aetiologies, including congenital without family history (5.4%), iatrogenic (1.7%), and perinatal hypoxia (0.3%) were relatively rare.

### Quality of life

The mean quality of life score was significantly higher in people without MSI (63.1, 95%CI 61.4–64.7) than among the cases (37.7, 35.4–40.0, p value<0.001. Severe cases had significantly poorer quality of life (16.9, 11.7–22.0) than moderate (34.9, 31.8–38.0), and mild cases (44.0, 40.5–47.8) (p-value 0.003).

### Treatment needed

In total, 641 treatments were needed for the 390 diagnoses (table 5). The most common treatments needed were physical therapy (44.5%), surgery (22.9%) or medication (16.1%). Extrapolating these estimates to the entire Rwandese population, approximately 814,000 treatments are required, including 356,000 courses of physical therapy, 184,000 operations and 129,000 courses of medicine.

**Table 5:** Treatment needed among cases with MSI in survey, and extrapolated to population of Rwanda.

Treatment modality	Number of cases in survey needing that treatment modality	Extrapolated number in country needing that treatment modality (based on 2005 population estimates) (95% CI)
Medication	103	132,000 (104,000–159,000)
POP / Splintage	53	68,000 (44,000–91,000)
Physical therapy	285	362,000 (340,000–383,000)
Mobility aid	12	15,000 (6,000–25,000)
Appliance	6	8,000 (2,000–14,000)
Orthosis	16	21,000 (10,000–30,000)
Prosthesis	5	6,000 (1,000–12,000)
Wheelchair	8	10,000 (3,000–17,000)
Surgery	147	187,000 (162,000–212,000)
Permanent care	6	5,000 (0–10,000)
TOTAL	641	814,000

## Discussion

This study estimates that the prevalence of MSI in Rwanda is 5.2% (95% CI 4.5–5.9). The prevalence of MSI is similar in men and women, but is much higher in older people as a result of an increase in cases caused by trauma, and degenerative changes (classified under the category ‘ acquired non traumatic non infective causes’), that are more prevalent in this age group. The overall prevalence for the population is higher than might be expected when compared to previous studies in Rwanda, but is in line with expectations of WHO and other prevalence estimations for other countries in the region [8], [9]. In addition to comparisons with other historical surveys and estimations, the accuracy of this survey can be measured against studies of common congenital abnormalities such as club foot. In this case the measured prevalence of 0.07% for club foot is consistent with an incidence of around 1 in 1000 live births as has been measured in other international studies [15].

53% of cases of MSI were moderate or severe according to the ICF classification, thus they significantly affect the life of the individuals concerned and their communities, and will have implications for the development of rehabilitation and other services in Rwanda.

The survey has produced results that will be of use in planning rehabilitation and other services in Rwanda. For example good estimations can be drawn as to the need for appliances, orthoses, prostheses and wheelchairs. This knowledge of need can be used as a target for production and supply of these items. Similarly accurate estimations of the need for medical services such as physiotherapy and surgery can be used to measure the capacity of existing services, and for advocacy and planning of future service provision. The estimations can be used to plan both building of medical facilities, and training of personnel such as physical therapists, orthotists, prosthetists, clinicians and surgeons, to treat the burden of musculoskeletal impairment.

Some MSIs are potentially preventable, in particular those involving trauma, and to a lesser extent infection. This survey was not intended to show how such prevention might be carried out, but it helps in planning as it gives an indication of the reduction in overall burden of disability in the community if particular MSIs can be prevented or at least reduced.

The study had many strengths, which lend confidence to the estimates obtained. A major strength is that a nationally representative sample of people of all ages was enumerated and examined. There is unlikely to have been serious selection bias, as the response rate was high and the sample was representative of the national population in terms of age- and sex- distribution. Furthermore, the reported prevalence of MSI among the sample and non-responders was very similar at 5.2% and 5.5% respectively. Information bias was also unlikely as outcome definition was undertaken by experienced physiotherapists, using a sensitive screening tool [13], and a robust questionnaire and examination protocol. There was good inter-observer agreement between the examiners and sensitivity and specificity of diagnosis was high. Furthermore, the specific diagnoses of MSI could be mapped on to ICF impairment categories for comparison with other ICF linked studies. The survey methodology was practical for use in a low-income country after a relatively short in-country training programme.

There were also limitations to the study. Since the examinations were carried out door-to-door, the diagnostic tools were limited to history and a clinical examination. It is also intentionally limited to MSI and does not give an estimate of other areas of impairment such as blindness, deafness or mental impairment. The in-country study costs were in the region of $100,000 and this may limit its use in other low income countries. This sum may seem high, but it reflects the real costs in mobilising a local survey team and giving logistic support over two months.

In conclusion, this survey demonstrates a large burden of MSI in Rwanda, which is mostly untreated. The demonstrated need will be useful in planning services. The survey methodology will also be useful in other low income countries.

## Supporting Information

Appendix S1 (0.03 MB DOC)
