# Supplementary material for: A National Survey of Musculoskeletal Impairment in Rwanda: Prevalence, Causes and Service Implications
Source: PLoS One. 2008 Aug 6;3(7):e2851. doi: 10.1371/journal.pone.0002851 (PMC2483936; doi:10.1371/journal.pone.0002851)
Supplement: Appendix S1 — (0.03 MB DOC) [file pone.0002851.s001.doc]

Appendix 1 – Design effect estimation

This study uses a clustered design. The participants in the survey are not independent individuals as the Primary Sampling Unit is not individuals but clusters. There are two sources of variation in a cluster; the variation between people in the same cluster and the variation between clusters (the combination of the two is the intra-cluster correlation coefficient; ICC). Clustering in the design of a study leads to larger standard errors, wider confidence intervals and larger P values than would be expected if simple randomisation of the participants had occurred. Calculating the sample size for a clustered design study requires multiplying the un-clustered (SRS) design sample size, by the design effect (DEFF) (19) thus:

Sample size _clustered_ = Sample size _unclustered_ **×** DEFF.

DEFFs are difficult to determine but can be estimated if there is a value for ICC:

DEFF = 1 + (r – 1)×ICC

A similar study to this one, looking at disability (20), had a DEFF of 2.2, which with the cluster size used gave an ICC of approximately 0.015. Using this ICC as an approximation, and with a chosen cluster size of 80 this gives us a design effect of 2.11.

**Appendix 2 – Outline of Sampling Methodology**

***Rwandan Population -*** Information from Rwandan 2002 Census

***Sampling by Probability Proportional to size***

Enumeration areas from the 2002 census of the population listed

Column of cumulative population created

***The Sampling Interval*** - Total population divided by number of clusters required (i.e. 105 from sample size calculation)

Sampling interval multiplied by random number between 0 and 1.

First ***Primary Sampling Unit (PSU)*** identified by tracing the resulting number in cumulative population column and taking the corresponding enumeration area

Following PSUs were identified by adding the sampling interval to the previous number.

***Enumeration Area - Primary Sampling Unit***

***In the Segment***

***Sampling by Compact Segment Sampling***

Enumeration Area Maps obtained from government (these depicted ***“nyumbakumi”*** – 10 household groups)

Enumerators update map nyumbakumi information

Enumeration Area population divided by to identify segments of 80 people

Segment chosen at random

Members of segment asked to remain (without being told nature of survey)

Household members only screened

80 people screened and identified as case/non-case

Screening stopped at 80 individuals regardless of whether the whole segment had been screened.

If whole segment screened and 80 not complete then random second segment screened until 80 is complete

Cases: all were assessed by the ***standardised examination protocol (SEP)*** section of the survey instrument

Random 10% of non-cases: assessed by the ***standardised examination protocol (SEP)*** thus continuing validation throughout the survey.

Sampling occurred without replacement to ensure field teams are motivated to seek out all respondents.

The sample size was inflated by 15% to allow for non-response.
